# Guillain-barré syndrome (GBS) with antecedent chikungunya infection: a case report and literature review

**DOI:** 10.1186/s42466-024-00315-6

**Published:** 2024-04-11

**Authors:** Sreelakshmi V., Amrita Pattanaik, Srilatha Marate, Reeta S Mani, Aparna R. Pai, Chiranjay Mukhopadhyay

**Affiliations:** 1 Manipal Institute of Virology, Manipal Academy of Higher Education, Manipal, 576104 Karnataka India; 2 Department of Neurovirology, National Institute of Mental Health and Neurosciences (NIMHANS), Karnataka Bengaluru, India; 3 Department of Neurology, Kasturba Medical College, Manipal Academy of Higher Education, Manipal, 576104 Karnataka India

**Keywords:** Guillain-Barré Syndrome, GBS, Fulminant, Antecedent infections, Arboviral infections, Chikungunya, Molecular mimicry, Intravenous immunoglobulin, AMSAN

## Abstract

Guillain-Barré Syndrome (GBS) is an autoimmune neuropathy. Antecedent infections have been seen to be significant triggering factors for developing GBS. Among them, arboviral infections are rapidly gaining importance as significant triggers, especially in the areas where they are endemic. Chikungunya, an arboviral infection that usually causes a self-limiting acute febrile illness can lead to GBS as one its severe complications. Herein, we describe a case of a 21-year-old female who presented with weakness in all four limbs and paresthesia. Nerve conduction study and cerebrospinal fluid (CSF) analysis showed axonal, demyelinating motor and sensory neuropathy with albuminocytological dissociation indicating Acute Motor and Sensory Axonal Neuropathy (AMSAN) variant of GBS. Serum IgM antibodies against ganglioside GM1 were detected. Anti-Chikungunya IgM antibodies were found in both serum and CSF samples. The patient was initiated with Intravenous Immunoglobulin (IVIG) therapy. In view of hypoxia, she was intubated and was on mechanical ventilation. After 2 weeks of being comatose, the patient gradually improved and was discharged with no sequelae.

A literature review on antecedent infections in GBS is presented alongside the case report to better understand the association of GBS with antecedent infections, especially the endemic arboviral infections like Chikungunya, Dengue and Zika. This will help in reinforcing the significance of having robust surveillance and public health control measures for infectious diseases.

## Background

Guillain-Barré Syndrome (GBS) is a rare but serious autoimmune disorder, affecting the peripheral nervous system (PNS). Highlighting the magnitude of the problem, globally, it has an annual incidence of 1–2 cases per 100,000 people [1]. However, regional differences in the incidence rate has been observed. As per the prevalence surveys done in Europe, Asia, America, and Australia, 0.4 to 4 GBS cases per 100,000 people have been reported annually [2]. In Asia, an annual incidence of 1.71, 1.82, 0.42 GBS cases per 100,000 people have been observed in China, South Korea, and Japan respectively [3, 4]. These cases have been more frequently reported during the monsoon season in some regions [2, 5]. However, contradicting this, in other geographical locations, studies have reported the peak of GBS cases in summer and winter, thus pointing towards a regional variation in the seasonality of the cases. Some studies also suggest that there is no discernible seasonal fluctuation [6, 7]. Increased incidence in GBS is also observed during outbreaks and pandemics. A recent instance is the significant rise in GBS cases following the COVID-19 pandemic [8–10]. Although the exact cause of GBS is still unknown, it is speculated to be a post-infectious condition since 2/3rd of the patients suffer from some form of infectious disease before the neurological condition sets in [11, 12]. Many bacterial and viral infections have been implicated in triggering the immune system against nerves, damaging it. As a consequence, weakness and tingling sensation in the extremities progressing to acute flaccid paralysis is seen [12, 13]. Less than one-third of the patients with GBS require mechanical ventilation due to respiratory muscle weakness. A mortality rate of 1–18% has been reported in such patients [14, 15]. Early diagnosis and treatment with intravenous immunoglobulin or plasmapheresis are effective in reducing the severity of the illness and aid in the recovery of the patients [12]. GBS is mostly a monophasic condition, but recurrence is observed in about 3–10% of the patients. It may occur at any age, however, higher incidences have been observed in adults compared to children with a predominance seen in males [16, 17].

GBS is a heterogeneous disorder having regional variation with respect to the clinical presentation, electrophysiological subtype, and outcome [18]. The lack of definitive cause and effective treatment is a major challenge that clinicians are facing even after 100 years since the reporting of the first case of GBS. Herein, we present a clinical case of fulminant GBS with antecedent Chikungunya infection. Chikungunya infection is an acute febrile illness usually associated with rashes and arthralgia, transmitted through *Aedes aegypti* and *Aedes albopictus* mosquitoes [19, 20]. Although meningitis, encephalitis, and GBS have been documented as consequences of a severe acute Chikungunya infection, neurological complications are uncommon [21]. Neurological complications of Chikungunya infection, including one case of GBS were first observed during the outbreak of 1964 in Madras, India [22].

The objectives of this review include: (i) reviewing the antecedent infections in GBS, (ii) illustrating the pathogenesis of GBS, (iii) describing the clinical features, and (iv) summarizing the treatment and management.

## Main text

### Case presentation

A 21-year-old female presented with rapid progressive weakness involving all four limbs associated with sensory symptoms in the form of paraesthesia followed by numbness. A month prior, she suffered from high-grade fever, chills, rigors with no other symptoms. There was no laboratory confirmed etiological diagnosis. No other premorbid conditions were found as well.

On initial examination, patient had respiratory distress with hypoxia, in view of which she was intubated and was put on control mode of ventilation. Neurological examination revealed tetraparesis, with paraesthesia of the extremities. There was bilateral but asymmetric facial palsy. Deep tendon reflexes were abolished. Pupils were sluggishly reactive. Routine blood examination revealed hyponatremia (126 mEq/L), anaemia with Vitamin B12 and folate deficiency, mild hypokalaemia (3.4 mmol/L), and elevated inflammatory markers. Nerve conduction study and CSF analysis identified axonal, demyelinating motor and sensory neuropathy with albuminocytological dissociation which indicated the Acute Motor Sensory Axonal Neuropathy (AMSAN) variant of GBS. Patient’s serum was positive for IgM antibodies against GM1 ganglioside; IgM antibodies against Chikungunya virus were found to be present in both serum and CSF. IgM antibodies against other prevalent arboviruses like Dengue, Zika, Japanese encephalitis virus, West Nile virus were not detected in both serum and CSF samples.

IVIG therapy (0.4 g/kg/day for 5 days) along with other supportive measures were immediately initiated in the patient. Nevertheless, she continued to deteriorate and 2 days later, ocular movements were lost with power in limbs worsening to zero. The EEG demonstrated severe encephalopathy with theta waves, suggestive of deep coma attributed to hyponatremia and sedation during intubation. She remained comatose with Glasgow Coma Scale score of 3 even after the completion of one course of IVIG therapy. Therefore, another cycle of IVIG therapy was administered. Despite a normal MRI report, a diagnosis of Bickerstaff brainstem encephalitis was considered because of the clinical picture that was presented.

After 10 days, the patient could move her lip, jaw, shoulders, proximal limbs and showed distal flickering. She was eventually removed from ventilatory support. Even with protective measures in place, due to facial weakness, exposure keratitis occurred and was managed by lid taping by the ophthalmology consultant. While intubated, the patient developed paralytic ileus for a few hours which was managed conservatively with bowel rest and intravenous fluid therapy. Physiotherapy and swallowing exercises were started along with iron, vitamin B12, and folate supplementation and continued after discharge. At discharge, she was hemodynamically stable with power of 4/5 in all four limbs, she was able to swallow semi- solids, and maintained saturation in room air.

## Literature review: antecedent infections in GBS

### Methodology

We conducted the literature search through multiple search engines such as PubMed, Scopus, Medline, Google Scholar, and ScienceDirect using the search terms: “Guillain-Barré Syndrome”, “GBS”, “autoimmunity”, “antecedent infection”, “bacteria”, “viruses”, “molecular mimicry”, “arboviruses in GBS”, “Chikungunya infection in GBS”, “intravenous immunoglobulins”, “plasmapheresis”. Thereafter, the search was limited to observational and interventional studies on Guillain-Barré Syndrome with antecedent infections. Only articles published in English language were considered. Additionally, relevant studies were identified through reference analysis. The articles available from 1st January 1990 until 31st December 2023, under the above-mentioned criteria were scanned and relevant studies were included in this narrative review. The results are presented under four headings: Antecedent infections causing GBS, Pathogenesis of GBS, Clinical manifestations and Diagnosis, and Treatment and Prognosis.

In addition, all the published cases of GBS with antecedent Chikungunya were included in this literature review. Up to 31st December, 2023, nine papers were published and a total of 33 cases have been described (Table 1). There was no gender prevalence, and the average age of patients studied was 51.

### Antecedent infections causing GBS

Though the definitive cause of GBS is still unknown, it has been noted that antecedent infections within two to four weeks were present in 2-3rd of the GBS patients before the onset of neurological symptoms [11, 12]. A wide spectrum of infectious agents including bacteria and viruses have been reported to cause GBS. Among them, the commonest agent is the *Campylobacter jejuni*, a Gram-negative bacteria causing gastroenteritis which accounts for 33% of GBS cases in the western countries, and 45–60% in China and Japan [11, 23–28]. Other bacteria like *Mycoplasma pneumoniae* and *Haemophilus influenzae* have also been reported to trigger GBS [11, 29].

Several viral infections have been associated with triggering GBS and have been reported worldwide. A case-control study conducted in Netherlands in 154 GBS patients reported antecedent infections with CMV, EBV, Parainfluenza 1 virus, Influenza A, Influenza B, Adenovirus, Herpes simplex virus (HSV), Varicella zoster virus (VZV) [11]. Another study conducted in China with 150 GBS patients reported antecedent infections with Influenza A virus, Influenza B virus, Hepatitis E virus (HEV), Hepatitis A virus (HAV), Dengue virus, Cytomegalovirus (CMV), Epstein- Barr virus (EBV), Herpes simplex virus (HSV), Varicella- zoster virus (VZV), and Rubella virus [29]. Cytomegalovirus has been found to be the most common viral infection which triggers the immune system leading to GBS [11, 29–31]. Following CMV, Epstein Barr virus is found to be the next common antecedent viral infection causing approximately 10% of total GBS cases [11, 29, 32]. GBS cases in children are mostly due to antecedent respiratory viral infections [33]. Antecedent infection with HEV, HAV, and Hepatitis B virus were also reported to cause GBS [34–36]. Rarely, infections with Adenovirus, Rubella were also observed as antecedent event in GBS patients [37].

Among the arboviruses, antecedent Zika virus infection has been widely studied in GBS. The 2013 Zika virus outbreak in French Polynesia was followed by an increased number of GBS cases [38]. The Zika virus epidemic that started in Brazil in 2016 and disseminated to 15 countries and territories increased the GBS global incidence rate 2.6 times [39, 40]. Other arboviral infections including Dengue, Chikungunya, and Japanese Encephalitis have also been implicated in the pathogenesis of GBS. In a multinational case-control study conducted in Brazil, Argentina, and Malaysia during 2017–2019 to understand the association of GBS with antecedent arboviral infections, it was found that 55% (27/49) of patients had recent infections. Arboviral infections caused due to Dengue and Chikungunya virus were found in 4% of these cases [41]. Another study conducted in Mexico to predict the incidence of GBS owing to arboviral infections reported antecedent Zika and Dengue infections in GBS but intriguingly, GBS was not associated with antecedent Chikungunya infection [42]. Among studies conducted in India, one done in the northern region reported 11.5% (3/26) antecedent Dengue infection in GBS [43]. In another study conducted in southern India, it was found that 79.3% of GBS patients studied had antecedent infections, of which viral infections with Chikungunya virus, Japanese Encephalitis virus, and Dengue virus were prominent [44].

While the occurrence of GBS following Chikungunya infection is uncommon, it is recognised as a potential trigger for GBS, particularly during outbreaks and in regions where the virus is endemic. During the Chikungunya outbreak at Reunion Island, France in 2005–2006, Lebrun et al. reported 2 cases of GBS with antecedent Chikungunya infection. In both the patients, Chikungunya infection was confirmed by the presence anti-Chikungunya IgM and IgG antibodies in serum and CSF [45–53]. Antecedent Chikungunya infections in GBS reported globally in the last two decades are depicted in **(**Table 1**)**.

**Table 1:** Characteristics of published cases of GBS with antecedent Chikungunya infection

Reference	Method and Year	Place	Number of cases	Age	Sex	GBS variant	Treatment	Outcome
Lebrun et al. (45)	Letter to the editor 2006	Reunion Island, France	2	51 and 48	Both females	AMSAN	IVIG	Recovered
Lemant et al. (46)	Case series 2005–2006	Reunion Island, France	1	55	Male	NR	IVIG	Recovered
Balavoine et al. (47)	Case series 2014	Martinique and Guadeloupe	13	Mean Age- 61	10 Males 3 females	7 AIDP, 2 AMSAN, 2 MFS, 2 PCB, 1 BE	IVIG, Plasmapheresis	11 Recovered, 2 died
Villamil-Gómez et al. (48)	Case report 2014	Columbia	1	77	Female	AIDP	IVIG	Recovered
Matos et al. (49)	Observational study 2014	Brazil	9	Mean Age- 44	5 Males, 4 females	7 AIDP, 2 AMAN	IVIG	Recovered
do Rosario et al. (50)	Case series 2016	Brazil	3	NR	NR	NR	IVIG	Recovered
Agarwal et al. (51)	Case report 2017	India	2	18 and 30	Both male	AMAN and AIDP	Plasmapheresis	Recovered
Mahto et al. (52)	Case report 2018	India	1	21	Female	NR	IVIG	Recovered
Hameed et al. (53)	Case report 2019	Pakistan	1	36	Female	NR	IVIG	Recovered

GBS- Guillain Barre Syndrome, AIDP- “Acute Inflammatory Demyelinating Polyneuropathy,” AMAN- “Acute Motor Axonal Neuropathy,” AMSAN- “Acute Motor and Sensory Axonal Neuropathy,” MFS- “Miller Fisher Syndrome,” “PCB- Pharyngeal Cervical Brachial,” BE- “Bickerstaff’s brainstem encephalitis,” IVIG-Intravenous Immunoglobulins, NR- Not reported

### Pathogenesis of GBS

**Fig. 1:**
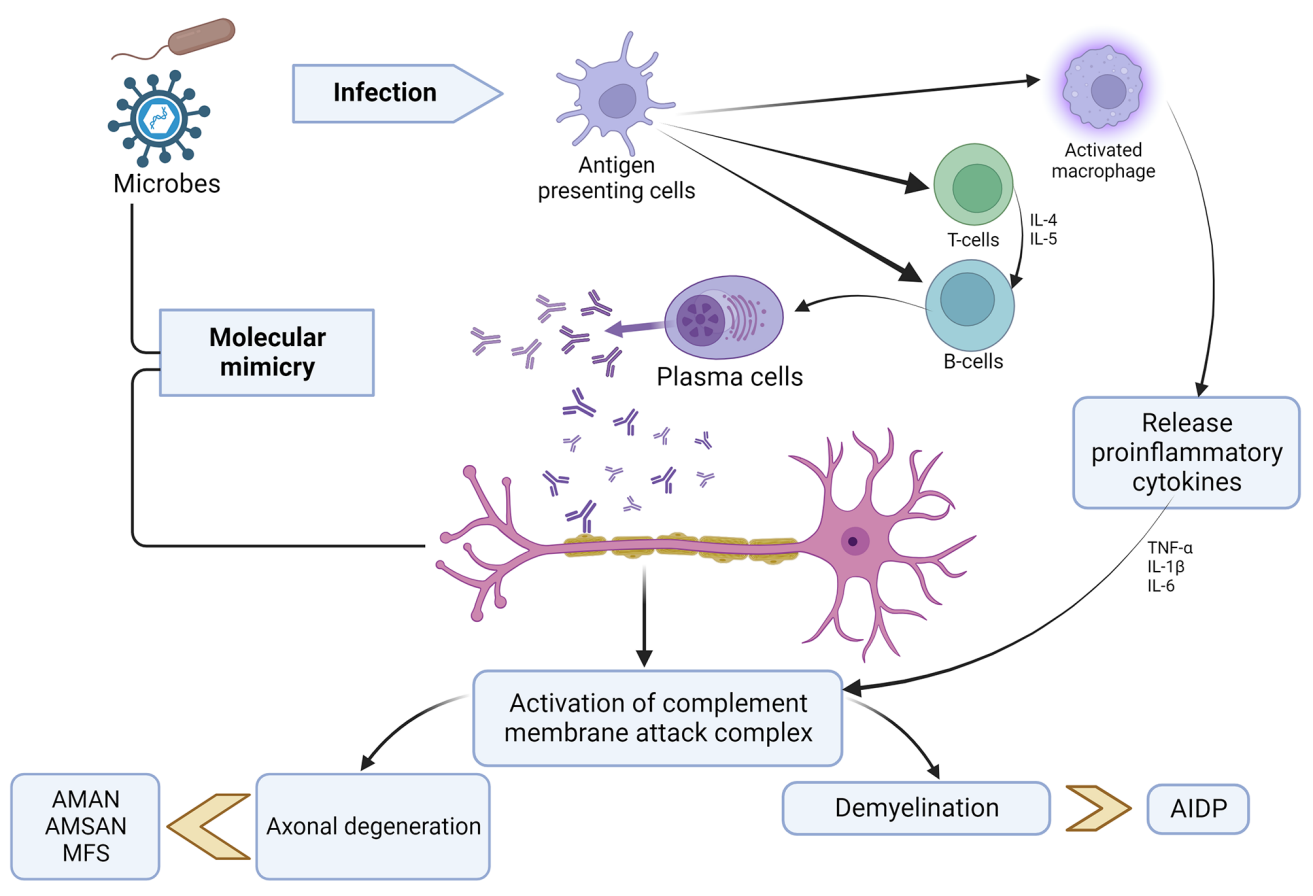
Pathogenesis of GBS

Th pathophysiology of GBS has been the subject of numerous studies, and research is still ongoing. The most accepted mechanism of pathogenesis is “molecular mimicry” at the B-cell level, particularly in the axonal variant **(**Fig. 1**)**. Previous studies found a structural similarity between lipopolysaccharide on the cell membrane of *C. jejuni* and the glycan molecule in the ganglioside, and the antibody produced against the bacteria led to nerve cell damage through cross-reactive immunological responses [54–56]. Supporting this, the animal studies carried out in “acute motor axonal neuropathy rabbit model” observed molecular mimicry in acute motor axonal neuropathy (AMAN) type of GBS [57]. Anti-ganglioside antibodies have been recorded in 36% of GBS patients [58]. These antibodies have been shown to have different peripheral nerve targets. Anti-GD1a antibodies bind to paranodal myelin, nodes of Ranvier, and neuromuscular junction. Anti-GM1 and anti-GQ1B antibodies bind to a peripheral nerve or neuromuscular junction [59]. This anti-ganglioside antibody mediated disease progression through complement cascade activation and formation of membrane attack complex plays a major role in the degeneration of nerve components [60].

AIDP- “Acute Inflammatory Demyelinating Polyneuropathy,” AMAN- “Acute Motor Axonal Neuropathy,” AMSAN- “Acute Motor and Sensory Axonal Neuropathy,” MFS- “Miller Fisher Syndrome,” TNF- “Tumor Necrosis Factor,” IL- “Interleukin”.

There are a few studies done in arboviral infections that have demonstrated anti-ganglioside antibodies. Dutta et al. observed an increase in anti-GD1a, GD1b, GT1b, and GQ1b antibodies in GBS patients with antecedent Japanese Encephalitis infection [61]. In a study conducted in immunocompetent mice, an increase in anti-GD1a and GD1b antibodies was observed in Zika virus associated GBS [62]. In another study involving GBS patients with antecedent Zika virus infection, antibody against GD3 ganglioside was found to be in high [63]. In GBS patients who had a prior COVID-19 infection, antibodies against GM1, GM2, GD1a, and GQ1b have been identified [64–66]. In the AIDP variant, autoantibodies against cell adhesion proteins like neurofascin, NRCAM, and contactin-2 localized at Ranvier’s nodes have been suggested as possible targets [67]. Similar evidence is not well documented for Chikungunya infection. Anti-ganglioside screening has been sparsely done in GBS cases associated with antecedent Chikungunya infection. Although molecular mimicry is speculated to be the most likely mechanism for such cases, there is a lack of evidence that demonstrates viral structure mimicking neural structures. There are two pathways of pathogenesis of Neuro-Chikungunya proposed. One is the direct viral CNS infection which may explain the quick onset of neurological symptoms in GBS patients with antecedent Chikungunya infection (as short as 2 or 3 days) and the second is the PNS affection by autoimmune mechanisms. However, there is a need to generate more evidence on it as well as on the possibility of viral mutations leading to neurological complications [68, 69].

Apart from anti-ganglioside antibodies, the epineurium and endoneurium of the nerves have demonstrated infiltration of T lymphocytes in GBS. CD4 + and CD8 + T lymphocytes, as well as macrophages, are observed in greater numbers. It is suggested that the activated T lymphocytes in infections release proinflammatory cytokines, that further activate the complement system leading to demyelination and axonal degeneration. TNF-α produced by the infiltrating T cells has a direct myelinotoxic effect on myelinated fibers, causing demyelination [70]. The exact mechanisms of pathogenesis, however, still remains unclear.

### Clinical manifestations and diagnosis

**Fig. 2:**
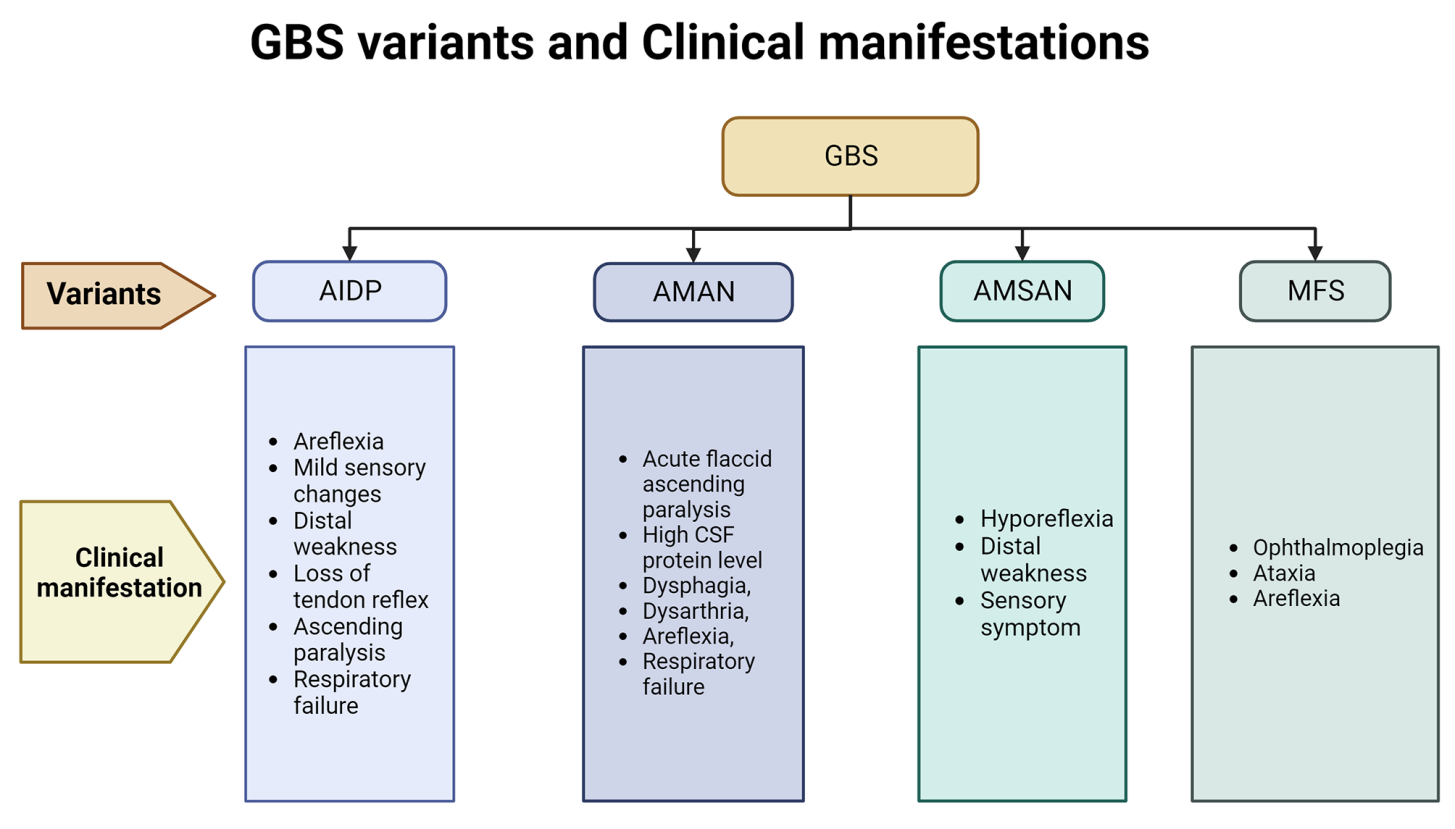
GBS variant and Clinical manifestation

GBS is caused by the autoimmune attack of peripheral nerves. After the onset of symptoms, it may take 24 hours to four weeks to reach the nadir. The clinical symptoms involved in GBS include weakness and tingling sensation of upper and/or lower limbs which may progress to paralysis of leg, arm, or facial muscles if left untreated [12].. Some patients may suffer from facial nerve palsy, oculomotor weakness, or oropharyngeal weakness. 10 to 30% GBS patients suffer from severe respiratory muscle weakness within 2 to 4 weeks and require mechanical ventilation [71]. Keesen et al. observed that in well-characterised GBS patients, respiratory involvement was more distinctively associated with antecedent Chikungunya infection as compared to other arboviral etiology [72]. Autonomic manifestations like ileus, urinary retention, fever, tachycardia or bradycardia, hypertension or hypotension are also reported in the GBS patients [73–75].. Fulminant GBS cases reported are characterized by the “absence of brainstem reflexes, complete tetraplegia, and respiratory arrest” [76]. Rarely, papilledema, facial myokymia, hearing loss, meningeal signs, change in mental status, vocal cord paralysis are also seen in GBS patients [77]. (Fig. 2) shows the clinical features seen in the different variants of GBS.

**Fig. 3:**
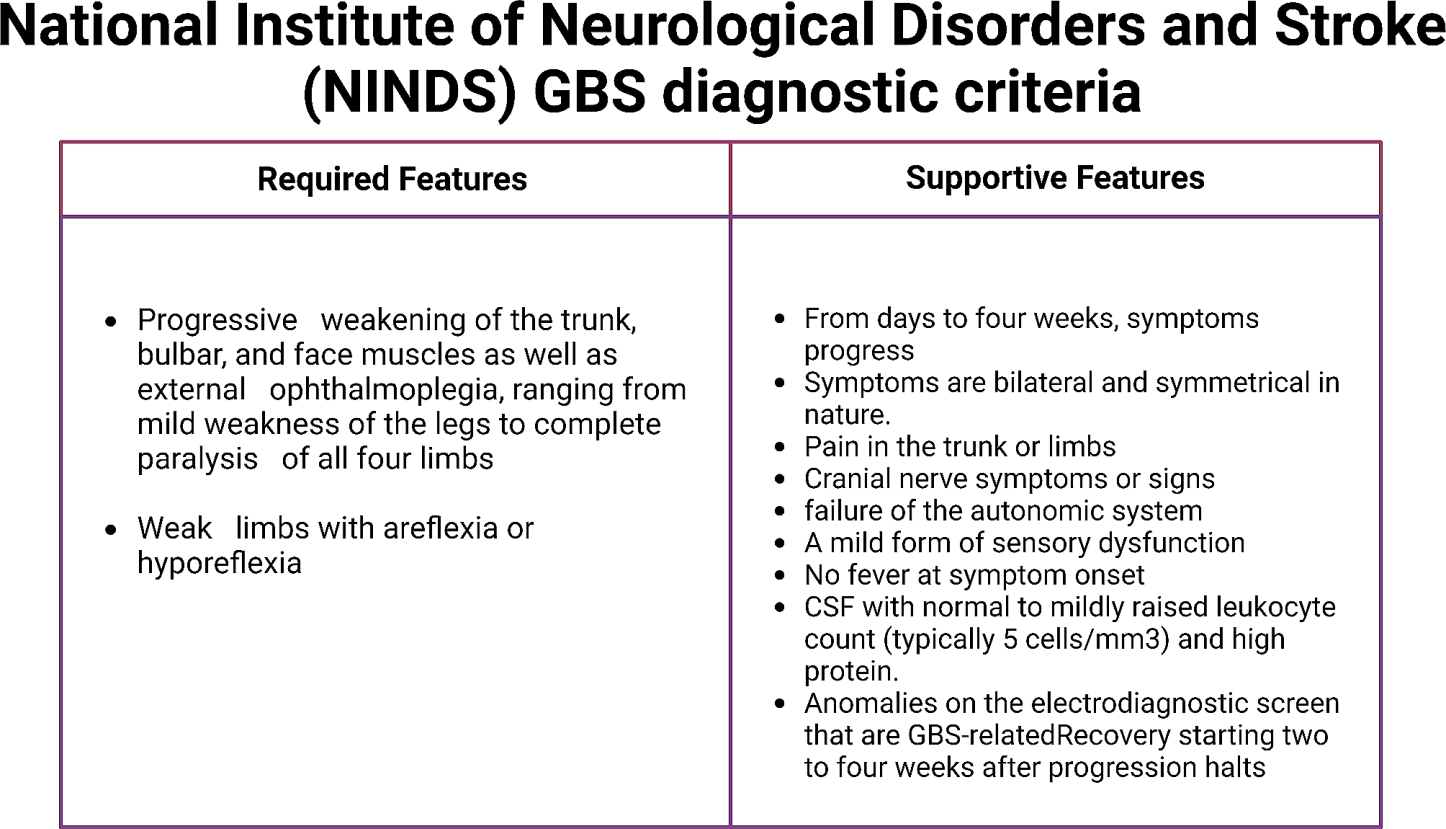
National Institute of Neurological Disorders and Stroke **(NINDS) GBS diagnostic criteria**

AIDP- “Acute Inflammatory Demyelinating Polyneuropathy,” AMAN- “Acute Motor Axonal Neuropathy,” AMSAN- “Acute Motor and Sensory Axonal Neuropathy,” MFS- “Miller Fisher Syndrome”.

A diagnosis of GBS is mostly based on the clinical history and physical examination. “National Institute of Neurological Disorders and Stroke (NINDS)” has published the GBS diagnostic criteria that is being widely followed (Fig. 3) [78]. The CSF examination and electrophysiological studies provide additional details about the condition. Routine laboratory investigations such as complete blood count, tests for blood glucose, electrolyte estimation, kidney and liver function tests are done in order to exclude other underlying pathologies. Anti-ganglioside antibodies found in the serum aid in identifying the GBS variant. In GBS, CSF protein level is elevated with a normal cell count. CSF examination is also done to find albumin-cytological dissociation [78]. The severity of the symptoms can be assessed by nerve conduction studies (NCS) and electromyography, which can aid in distinguishing between the axonal and demyelinating forms. Radiological studies can aid in cases where CSF and nerve conduction studies are inconclusive. Spine Magnetic Resonance Imaging (MRI) can exclude other conditions like compressive polyradiculopathy and transverse myelitis [79].

### Treatment and prognosis

Primarily IVIg (0.4 g/kg for 5 days) and plasma exchange (200–250 ml plasma/kg body weight in five sessions) are the recommended treatments for GBS which are quite effective. Due to better availability and ease of administration, IVIg is preferred over plasma exchange. Despite therapy, there is a significant mortality of 3–10% [78, 80].

ICU admission is warranted in about 22% of the patients with GBS due to increasing respiratory insufficiency, as was the case here; severe dysfunction in swallowing, cardiac abnormalities and in rapid progression of weakness. Respiratory distress requiring mechanical ventilation has been reported in cases with antecedent Chikungunya infection. In such cases, both acute and long-term mortality are low [78, 81].

Antiviral treatment can be considered in patients with GBS who have an ongoing treatable viral infection; however, preceding infections have usually resolved before the onset of weakness [78]. Arboviral infections including Chikungunya infection is managed by supportive measures. A few novel antiviral compounds and monoclonal antibodies are being evaluated against Chikungunya virus [82–84]..

Severe disability is seen in 20% of cases which require neurorehabilitation. Some of the delayed complications of GBS include neuropathic pain, postural hypotension, and fatigue. These may persist for months together [80].

## Conclusion

In conclusion, although GBS is considered as a rare disease, there is a perceptible increase in the incidence, especially during outbreaks and pandemics of infectious disease. Many studies mentioned in the literature review have concurred the same. Here we have reported a case of GBS associated with antecedent Chikungunya infection. Chikungunya infection most often causes a self-limiting disease; however, severe complications have been reported occasionally. The case described in this paper adds to the few cases of Chikungunya infection preceding GBS reported globally. This will help physiciansunderstand the clinical course and managesuch cases better. As per the evidence in literature, it is worth noting that acute Arboviral infections including Chikungunya infection lead to GBS in a short period of time that usually ranges from a few days to a few weeks. Most of these cases require ICU support due to frequently associated respiratory insufficiency. Documentation of such cases can help public health policy makers to undertake more surveillance programs and implement appropriate control measures to reduce the burden of Arboviral infections. Along with it, an understanding of the pathogenesis may lead to the development of more directed preventive and therapeutic interventions.

## Data Availability

Not applicable.

## Declarations

### Authors’ information

Manipal Institute of Virology, Manipal Academy of Higher Education, Manipal, Karnataka, India.

Sreelakshmi V, Amrita Pattanaik, Srilatha Marate, Chiranjay Mukhopadhyay.

Department of Neurovirology, National Institute of Mental Health and Neurosciences (NIMHANS), Bengaluru, Karnataka, India.

Reeta S Mani.

Department of Neurology, Kasturba Medical College, Manipal Academy of Higher Education, Manipal, Karnataka, India.

Aparna R Pai.

## Abbreviations

*GBS*: Guillain Barré Syndrome
*AIDP*: Acute Inflammatory Demyelinating Polyneuropathy
*AMAN*: Acute Motor Axonal Neuropathy
*AMSAN*: Acute Motor and Sensory Axonal Neuropathy
*MFS*: Miller Fisher Syndrome
*PCB*: Pharyngeal Cervical Brachial
*BE*: Bickerstaff’s brainstem encephalitis
*IVIG*: Intravenous Immunoglobulin
*LMN*: Lower Motor Neuron
*MRI*: Magnetic Resonance Imaging
*ET*: Endotracheal tube
*EEG*: Electroencephalogram
*PCR*: Polymerase Chain Reaction
*APC*: Antigen-presenting cells
*CMV*: Cytomegalovirus
*EBV*: Epstein Barr virus
*CD*: Cluster of differentiation
*RT-PCR*: Real-time reverse transcriptase polymerase chain reaction
